# Molecular and functional analyses of COPT/Ctr-type copper transporter-like gene family in rice

**DOI:** 10.1186/1471-2229-11-69

**Published:** 2011-04-21

**Authors:** Meng Yuan, Xianghua Li, Jinghua Xiao, Shiping Wang

**Affiliations:** 1 National Key Laboratory of Crop Genetic Improvement, National Center of Plant Gene Research (Wuhan), Huazhong Agricultural University, Wuhan 430070, China

## Abstract

**Background:**

The copper (Cu) transporter (COPT/Ctr) gene family has an important role in the maintenance of Cu homeostasis in different species. The rice COPT-type gene family consists of seven members (*COPT1 *to *COPT7*). However, only two, *COPT1 *and *COPT5*, have been characterized for their functions in Cu transport.

**Results:**

Here we report the molecular and functional characterization of the other five members of the rice *COPT *gene family (*COPT2*, *COPT3*, *COPT4*, *COPT6*, and *COPT7*). All members of the rice *COPT *family have the conserved features of known *COPT/Ctr*-type Cu transporter genes. Among the proteins encoded by rice *COPTs*, COPT2, COPT3, and COPT4 physically interacted with COPT6, respectively, except for the known interaction between COPT1 and COPT5. COPT2, COPT3, or COPT4 cooperating with COPT6 mediated a high-affinity Cu uptake in the yeast *Saccharomyces cerevisiae *mutant that lacked the functions of ScCtr1 and ScCtr3 for Cu uptake. COPT7 alone could mediate a high-affinity Cu uptake in the yeast mutant. None of the seven COPTs alone or in cooperation could complement the phenotypes of *S. cerevisiae *mutants that lacked the transporter genes either for iron uptake or for zinc uptake. However, these *COPT *genes, which showed different tissue-specific expression patterns and Cu level-regulated expression patterns, were also transcriptionally influenced by deficiency of iron, manganese, or zinc.

**Conclusion:**

These results suggest that COPT2, COPT3, and COPT4 may cooperate with COPT6, respectively, and COPT7 acts alone for Cu transport in different rice tissues. The endogenous concentrations of iron, manganese, or zinc may influence Cu homeostasis by influencing the expression of *COPTs *in rice.

## Background

Copper (Cu) is an essential micronutrient for living organisms. Cu, as a cofactor in proteins, is involved in a wide variety of physiological processes. Cu has an impact on the development of the nervous system in animals and humans; deficiency of this micronutrient causes Menkes syndrome in humans [1,2]. In plants, Cu is associated with various physiological activities, such as photosynthesis, mitochondrial respiration, superoxide scavenging, cell wall metabolism, and ethylene sensing [3]. Cu deficiency causes diverse abnormal phenotypes in plants, including decreased growth and reproductive development, distortion of young leaves, and insufficient water transport [4]. Cu can also be a toxic element when present in excess by generating hydroxyl radicals that damage cells at the level of nucleic acids, proteins, and lipids or by reacting with thiols to displace other essential metals in proteins [4]. Wilson disease in humans is caused by the accumulation of Cu in the liver and brain [1]. The most common symptom of Cu toxicity in plants is chlorosis of vegetative tissues due to the dysfunction of photosynthesis [4].

To deal with this dual nature of Cu, plants, as well as other organisms, have developed a sophisticated homeostatic network to control Cu uptake, trafficking, utilization, and detoxification or exportation [5,6]. A main step in the control of Cu homeostasis is its uptake through the cell membrane. Different types of transporter proteins that can mediate Cu uptake have been reported. The major group is the COPT (COPper Transporter)/Ctr (Copper transporter) proteins, which belong to multiple protein families in different organisms [7]. The P-type adenosine triphosphate pump is another type of transporter for moving Cu from cytosol into organelles in humans and plants [8,9]. It has also been reported that other metal transporters can transport Cu into cells. For example, YSL1 and YSL3 of the OPT/YSL iron (Fe) transporter family can transport Cu from leaves to seeds in Arabidopsis [10], and ZIP2 and ZIP4 of the ZIP zinc (Zn) transporter family seem to transport Cu in Arabidopsis [11].

The Cu-uptake function of COPT/Ctr proteins was described primarily in baker's yeast, *Saccharomyces cerevisiae *[12]. Soon afterward, COPT/Ctr proteins involved in Cu transport were characterized in different organisms, for example: ScCtr1, ScCtr2, and ScCtr3 in *S. cerevisiae *[12-15]; AtCOPT1, AtCOPT2, AtCOPT3, AtCOPT4, and AtCOPT5 in *Arabidopsis thaliana *[13,16]; hCtr1 and hCtr2 in humans (*Homo sapiens*) [17,18]; SpCtr4, SpCtr5, and SpCtr6 in fission yeast (*Schizosaccharomyces pombe*) [19-21]; mCtr1 in mouse (*Mus musculus*) [22]; PsCtr1 in lizard (*Podarcis sicula*) [23]; Ctr1A, Ctr1B, and Ctr1C in *Drosophila melanogaster *[24]; DrCtr1 in bony fish (*Danio rerio*) [25]; CaCtr1 in ascomycete *Colletotrichum albicans *[26]; CgCtr2 in ascomycete *C. gloeosporioides *[27]; CrCTR1, CrCTR2, CrCTR3, and CrCOPT1 in green algae (*Chlamydomonas reinhardtii*) [28]; and OsCOPT1 and OsCOPT5 in rice (*Oryza sativa*) [29]. COPT/Ctr-like proteins also occur in zebrafish [15], ascomycete *Neurospora crassa *[30], and mushroom *Coprinopsis cinerea *[31], although their functions in Cu uptake remain to be determined.

The alignment of COPT/Ctr family members from different species highlights domains that are structurally conserved and probably functionally important during the evolution of these proteins. According to bioinformatic analyses, all COPT/Ctr proteins contain three putative transmembrane regions [7]. Characterized COPT/Ctr proteins are either plasma membrane proteins that transport Cu from extracellular spaces into cytosol or vacuoles or lysosome membrane proteins that deliver Cu from vacuoles or lysosomes to the cytosol [15,18,20,32]. COPT/Ctr proteins can form a homodimer, homotriplex [22,33], or heterocomplex with themselves or each other [19,21] or form a heterocomplex with another protein that is associated with Cu transport [29]. A structural model has been proposed for the function of human homotrimeric hCtr1 in which Cu(I) first coordinates to the methionine-rich motif in the hCtr1 extracellular amino (N)-terminus and then a conserved methionine residue upstream of the first transmembrane domain is essential for Cu transport [33,34]. The homotriplex is thought to provide a channel for passage of Cu across the lipid bilayer [35]. The conserved paired cysteine residues in the cytoplasmic carboxyl (C)-terminus of yeast ScCtr1 serve as intracellular donors for Cu(I) for its mobilization to the Cu chaperones [36].

Studies have revealed that the expression of *COPT/Ctr *genes is controlled by environmental Cu level in different species [7,29,37]. In general, they are transcriptionally up-regulated in response to Cu deprivation and down-regulated in response to Cu overdose. The functions of COPT/Ctr proteins are also influenced by other factors. For example, the human hCtr1-mediated Cu transport is stimulated by extracellular acidic pH and high K^+ ^concentration [22].

The COPT family of rice (*Oryza sativa *L.) consists of seven members, COPT1 to COPT7. COPT1 and COPT5 can form homodimers or a heterodimer. The two COPTs, but not other rice COPTs, bind to different sites of rice XA13 protein, which is a susceptible protein to pathogenic bacterium *Xanthomonas oryzae *pv. *oryzae *(*Xoo*) [29]. Expression of COPT1, COPT5, or XA13 alone or coexpression of any two of the three proteins could not complement the phenotype of yeast *S. cerevisiae *mutant, which lacked the functions of ScCtr1 and ScCtr3 for Cu uptake; only coexpression of all three proteins complemented the mutant phenotype [29]. However, it is unknown whether rice COPT2, COPT3, COPT4, COPT6, and COPT5 are also involved in Cu transport. In this study, we analyzed the putative Cu-uptake functions of the five rice COPTs using a yeast *ctr *mutant. We also analyzed the spatiotemporal, tissue-specific, metal-responsive, and pathogen-responsive expression patterns of rice *COPTs*. The results suggest that rice *COPTs *are transcriptionally influenced by multiple factors. Except for COPT1 and COPT5, other COPTs may function alone or cooperatively to mediate Cu transport in different rice tissues.

## Methods

### Plant treatment

To study the effect of Cu on gene expression, four-leaf seedlings of rice variety Zhonghua 11 (*Oryza sativa *ssp. *japonica*) were grown in hydroponic culture containing standard physiological Cu (0.2 mM), manganese (Mn; 0.5 mM), Fe (0.1 mM), and Zn (0.5 mM) or overdose Cu (50 mM) as described previously [29]. To induce a deficiency of Cu, Mn, Fe, or Zn, plants were grown in the culture media lacking Cu, Mn, Fe, or Zn for 2 weeks.

To analyze the effect of bacterial infection on gene expression, plants were inoculated with Philippine *Xoo *strain PXO61 (race 1) or PXO99 (race 6) at the booting (panicle development) stage by the leaf-clipping method [38]. The 2-cm leaf fragments next to bacterial infection sites were used for RNA isolation.

### Gene expression analysis

Gene expression was analyzed as described previously [29]. In brief, total RNA was isolated from different tissues of rice variety Zhonghua 11 using TRIzol (Life Technologies Corporation, Carlsbad, CA, USA) according to the manufacturer's instruction. The concentration of RNA was measured with a NANODROP 1000 Spectrophtometer (Thermo Scientific, Wilmington, DE, USA); the A260/A280 ratio was generally between 1.8 and 2.0; RNA concentration was approximately 60 μg/100 mg fresh rice tissue. An aliquot (5 μg) of RNA was treated with 1 unit of DNase I (Life Technologies Corporation) in a 20-μl volume for 15 minutes to remove contaminating DNA and then used for quantitative reverse transcription-polymerase chain reaction (qRT-PCR) analysis. The RT step was performed in a 20-μl volume containing 5 μg of RNA, 100 ng oligo(dT)_15 _primer, 2 nmol dNTP mix, and 100 unit of M-MLV reverse transcriptase (Life Technologies Corporation) according to the manufacturer's instruction. The cDNA was stored at -20°C. The qPCR was performed using the SYBR Premix Ex Taq kit (TaKaRa Biotechnology, Dalian, China) on the ABI 7500 Real-Time PCR system (Applied Biosystems, Foster City, CA, USA). Gene-specific PCR primers are listed in Additional file 1, Table S1. The expression level of actin gene was first used to standardize the RNA sample for each qRT-PCR. The expression level relative to control was then presented. Each qRT-PCR or RT-PCR assay was repeated at least twice with similar results, with each repeat having three replicates.

### Plasmid constructs

The full-length cDNAs of rice *COPT2*, *COPT3*, *COPT4*, *COPT6*, and *COPT7 *genes were obtained by RT-PCR using gene-specific primers (Additional file 1, Table S2). The cDNAs of *COPT2*, *COPT3*, and *COPT6 *were subcloned into the *Bam*HI/*Eco*RI sites of p413GPD(+His) vector and the cDNAs of *COPT4 *and *COPT7 *were subcloned into the *Spe*I/*Eco*RI sites of p413GPD(+His) separately [39]. The cDNA of *S. cerevisiae Ctr1 *was amplified from yeast BY4741 using gene-specific primers (Additional file 1, Table S2) and was inserted into the *Spe*I/*Eco*RV sites of p413GPD(+His). All the cDNA sequences were confirmed by DNA sequencing. The p413GPD(+His) plasmids carrying respective *COPT1 *and *COPT5 *were as reported previously [29].

### Functional complementation analyses in yeast

To study the putative function of genes in Cu transport, plasmid DNA was transformed into the yeast *ctr1Δctr3Δ *double-mutant strain MPY17 (*MATα*, *ctr1::ura3::kan*^*R*^, *ctr3::TRP1*, *lys2-801*, *his3*), which lacked the Ctr1 and Ctr3 for high-affinity Cu uptake, by the lithium acetate procedure [14]. This mutant strain cannot grow on ethanol/glycerol medium (YPEG: 1% yeast extract, 2% Bactopeptone, 2% ethanol, 3% glycerol, 1.5% agar) because it possesses a defective mitochondrial respiratory chain due to the inability of cytochrome *c *oxidase to obtain its Cu factor [14]. The transformed yeast cells were grown in SC-His to OD_600nm _= 1.0. Several 10-fold diluted clones were plated as drops on selective media without or with supplement of Cu (CuSO_4_). Plates were incubated for 3 to 6 days at 30°C.

To study the putative functions of genes in Fe or Zn transport, plasmid DNA was transformed into the yeast *fet3fet4*DEY1453 or *zrt1zrt2*ZHY3 mutants or the wild-type strain DEY1457 by the same lithium acetate procedure described above. The *fet3fet4*DEY1453 double mutant (MAT*α trp1 ura3 *Δ*fet3*::*LEU2 *Δ*fet4*::*HIS3*) lacked the Fet3 and Fet4 for Fe uptake [40]. The *zrt1zrt2*ZHY3 double mutant (MAT*α ade6 can1 his3 leu2 lys2 trp1 ura3 zrt1*::*LEU2 zrt2*::*HIS3 *Δ*fet3*::*LEU2 *Δ*fet4*::*HIS3*) lacked the Zrt1 and Zrt2 for Zn uptake [41,42]. Yeast cells were grown in YPD (yeast extract-peptone) medium (1% yeast extract, 2% peptone, 2% glucose) to OD_600nm _= 1.0. Several 10-fold diluted clones were plated as drops on selective media containing 50 mM bathophenanthroinedisulfonic acid disodium (BPDS) without or with supplement of Fe (FeSO_4_), or containing 1 mM EDTA without or with supplement of Zn (ZnSO_4_). BPDS was a synthetic chelate of Fe and EDTA was a synthetic chelate of Zn. Plates were incubated for 3 to 6 days at 30°C. The yeast strains transformed with empty vector were the negative controls.

### Protein-protein interaction in yeast cells

The split-ubiquitin system was used to investigate the interaction of rice COPTs. The yeast two-hybrid (Y2H) Membrane Protein System Kit (MoBiTec, Goettingen, Germany) was used for this type of assay according to the manufacturer's instructions. In this system, COPT proteins fused with both the C-terminal half of the ubiquitin protein (Cub) and the mutated N-terminal half of the ubiquitin protein (NubG). Cub could not interact with NubG. When COPT proteins interacted, Cub and NubG were forced into close proximity, resulting in the activation of reporter gene. Each *COPT *full-length cDNA amplified from rice variety Zhonghua 11 using gene-specific primers (Additional file 1, Table S3) was cloned into both vector pBT3-SUC and vector pPR3-SUC; the 3' ends of *COPT *cDNAs were fused with the 5' end of the sequence encoding NubG. The insertion fragments of all the vectors were examined by DNA sequencing. Cub and NubG fusion constructs were co-transformed into host yeast strain NMY51. Interaction was determined by the growth of yeast transformants on medium lacking His or Ade and also by measuring β-galactosidase activity.

### Protein topology analyses

The localization of rice COPT2, COPT3, or COPT4 protein in *S. cerevislae *was analyzed by fusion of the *COPT *gene with the green fluorescence protein (GFP) gene using gene-specific primers (Additional file 1, Table S4). Plasmid harboring the fusion gene was transformed into the *S. cerevislae *mutant MPY17 [29]. The fluorescence signal was visualized with a LEICA DM4000B fluorescent microscope.

### Sequence analysis

Multiple-sequence alignment of amino acid sequences was achieved with ClustalW program http://www.expasy.ch/tools/#align[43]. A neighbor-joining phylogenetic tree was constructed in the ClustalX program based on the full sequences of the proteins with default parameters [44]. The transmembrane domains of proteins were predicted using the TMHMM program http://www.cbs.dtu.dk/services/TMHMM/.

## Results

### Rice COPT family has all the conserved features of known COPT/Ctr-type Cu transporter genes

**Figure 1:**
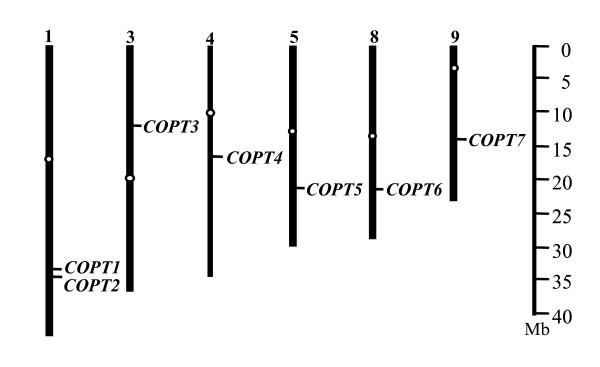
**Chromosomal locations of rice *COPT *genes**. The centromere of each chromosome is indicated with a white circle.

Analysis of the genomic sequences of rice variety Nipponbare (*Oryza sativa *ssp. *japonica*) revealed seven genes, *COPT1 *(Os01g56420; GenBank accession number GQ387494), *COPT2 *(Os01g56430; HQ833653), *COPT3 *(Os03g25470; HQ833654), *COPT4 *(Os04g33900; HQ833655), *COPT5 *(Os05g35050; GQ387495), *COPT6 *(Os08g35490; HQ833656), and *COPT7 *(Os09g26900; HQ833657), that showed sequence homology with *COPT *or *Ctr *genes from other species [29]. The seven *COPTs *are located on six of the 12 rice chromosomes (1, 3, 4, 5, 8, and 9) (Figure 1). Based on the annotation of Rice Genome Annotation Project (RGAP; http://rice.plantbiology.msu.edu/), all seven genes are intron-free. The seven genes are G- and C-rich consisting more than 72% G and C.

**Figure 2:**
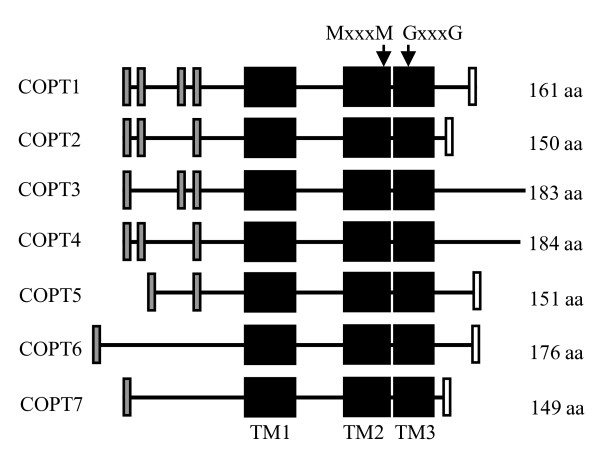
**The structure of rice COPT proteins**. Lines represent the protein chains. Black boxes represent predicted transmembrane (TM1, TM2, TM3) regions. Gray boxes indicate the positions of MxM or MxxM motifs and white boxes indicate the positions of CC or CxC motifs.

**Figure 3:**
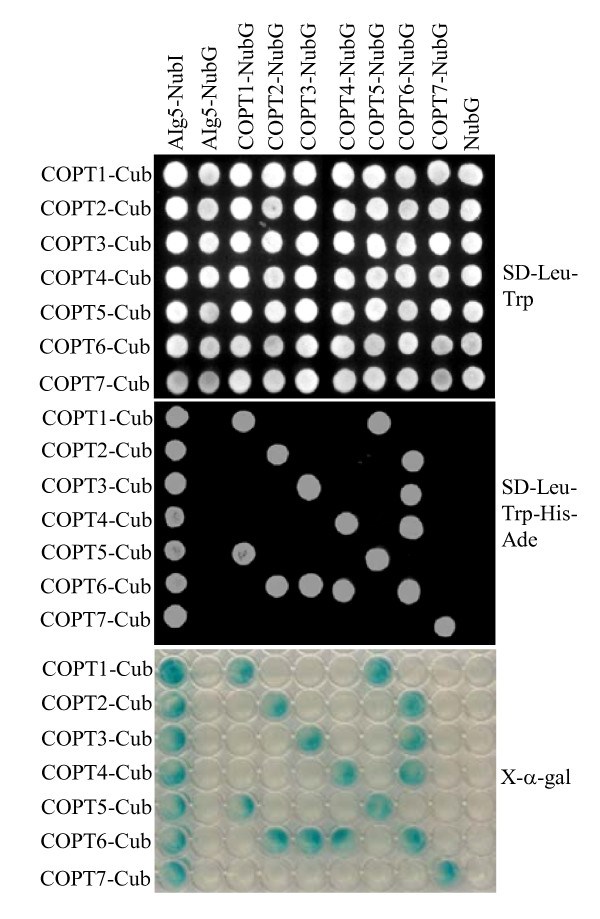
**Analyses of the interactions of rice COPTs protein by split-ubiquitin system**. The growth of yeast cells on selective medium and the expression of reporter protein a-galactosidase (X-a-gal) indicate the homomeric or heteromeric interactions of COPTs. AIg5, a transmembrane protein with C terminal in the cytoplasm; NubI, N-terminal half of ubiquitin protein; NubG, mutated N-terminal half of ubiquitin protein; Cub, C-terminal half of ubiquitin protein; Leu, leucine; Trp, tryptophan; His, histidine; Ade, adenine.

The rice COPT proteins share 35% to 64% sequence identity and 47% to 73% sequence similarity each other (Table 1) and have a similar structure (Figure 2), which is similar to COPT/Ctr proteins in other species [11]. The central domains of rice COPTs are three transmembrane (TM) regions, TM1, TM2, and TM3 (Figure 2). TM1 and TM2 in the COPT/Ctr proteins of other species are separated by a cytoplasmic loop [7,45]. Only a few residues separate TM2 and TM3 of rice COPTs (Figure 2). It has been reported that a short loop connecting TM2 and TM3 is essential for function and it enforces a very tight spatial relationship between these two TM segments at the extracellular side of plasma membrane [46]. Rice TM2 harbors the MxxxM (x representing any amino acid) motif and TM3 contains the GxxxG motif, together forming the characteristic MxxxM-x_12_-GxxxG motif of COPT/Ctr proteins [46]. COPT/Ctr proteins usually have an extracellular N-terminus and a cytoplasmic C-terminus; the N-terminus is generally rich in conserved methionine-rich motifs, which are important for low level of Cu transport; the C-terminus tends to contain cysteine- and/or histidine-rich motifs, such as CxC, which binds Cu ions and transfers them to cytosolic Cu chaperones [45,47]. All these key features are present in rice COPTs. COPT1 and COPT5 have cytoplasmic C-termini [29]. The interactions of COPT2, COPT3, COPT4, COPT6, and COPT7 with Alg5, a TM protein, in yeast cells using a split-ubiquitin system for integral membrane proteins [48] suggests that the other five rice COPTs also have cytoplasmic C-termini (Figure 3). All seven COPTs have one to four MxM or MxxM motifs at N-termini (Figure 2). The CC motif has been detected in rice COPT1, COPT2, and COPT5 and CxC motif in rice COPT6 and COPT7 at the C-termini (Figure 2).

**Table 1:** Analysis of amino acid sequence identity/similarity (%) among members of rice COPT family

	COPT1	COPT2	COPT3	COPT4	COPT5	COPT6	COPT7
COPT1	100/100	64/71	47/54	49/57	64/71	58/62	37/51
COPT2		100/100	48/56	45/53	64/73	60/66	38/50
COPT3			100/100	59/61	50/58	57/61	56/67
COPT4				100/100	54/61	60/62	36/47
COPT5					100/100	56/64	35/47
COPT6						100/100	50/60
COPT7							100/100

**Figure 4:**
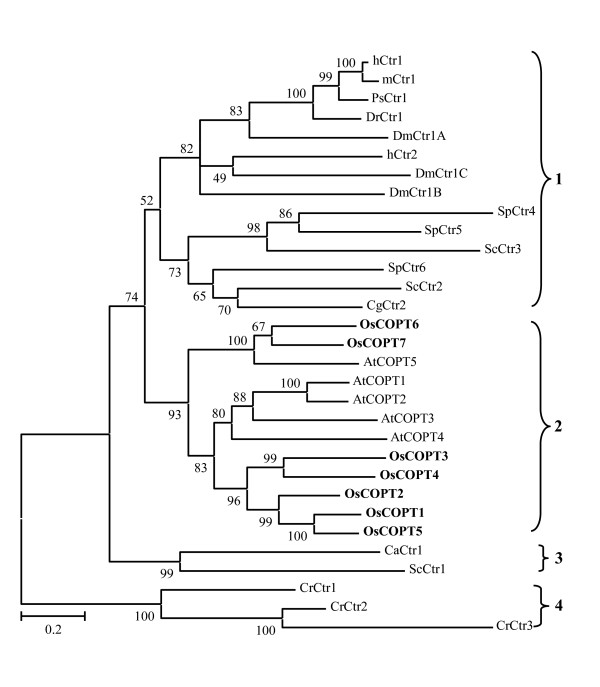
**Phylogenetic relationship of COPT/Ctr proteins from different species**. The COPT/Ctr proteins were from human (hCtr1, accession number of GenBank or Protein database of National Center for Biotechnology Information http://www.ncbi.nlm.nih.gov: NP_001850; hCtr2, NP_001851), mouse (mCtr1, NP_780299), lizard (PsCtr1, CAD13301), zebrafish (DrCtr1, NP_991280), fruit fly (DmCtr1A, NP_572336; DmCtr1B, NP_649790; DmCtr1C, NP_651837), baker's yeast (ScCtr1, NP_015449; ScCtr2, NP_012045; ScCtr3, NP_013515), fission yeast (SpCtr4, NP_587968; SpCtr5, NP_594269; SpCtr6, NP_595861), *Colletotrichum gloeosporioides *(CgCtr2, ABR23641), Arabidopsis (AtCOPT1, NP_200711; AtCOPT2, NP_190274; AtCOPT3, NP_200712; AtCOPT4, NP_850289; AtCOPT5, NP_197565), *C. albicans *(CaCtr1, CAB87806), and green algae (CrCtr1, XP_001693726; CrCtr2, XP_001702470; CrCtr3, XP_001702650). The numbers for interior branches indicate the bootstrap values (%) for 1000 replications. The scale at the bottom is in units of number of amino acid substitutions per site.

A phylogenetic analysis was performed to examine the evolutionary relationship of 31 COPT/Ctr proteins among rice and other species. This analysis classified these proteins into four groups (Figure 4). Group 1 includes Ctr proteins from humans (hCtr1 and hCtr2), mouse (*Mus musculus*; mCtr1), lizard (*Podarcis sicula*; PsCtr1), zebrafish (*Danio rerio*; DrCtr1), fruit fly (*Drosophila melanogaster*; DmCtr1A, DmCtr1B, DmCtr1C), baker's yeast (ScCtr2 and ScCtr3), fission yeast (*S. pombe*; SpCtr4, SpCtr5, and SpCtr6), and ascomycetes (*Colletotrichum gloeosporioides*; CgCtr2). Group 2 consists of seven rice COPTs (*Oryza sativa*; OsCOPT1 to OsCOPT7) and five Arabidopsis COPTs (*Arabidopsis thaliana*; AtCOPT1 to AtCOPT5). Group 3 includes two Ctrs from ascomycetes (*C. albicans*; CaCtr1) and baker's yeast (ScCtr1), respectively. Group 4 consists of three Ctrs from green algae (*Chlamydomonas reinhardtii*; CrCtr1, CrCtr2, and CrCtr3). This analysis suggests that rice COPTs are evolutionarily related to Arabidopsis COPTs rather than other COPT/Ctr proteins. Furthermore, rice COPT6 and COPT7 are more related to Arabidiopsis COPT5; rice COPT1 to COPT5 are more related to Arabidopsis COPT1 to COPT4 (Figure 4).

### Rice COPTs function alone or cooperatively as high-affinity Cu transporters in yeast

**Figure 5:**
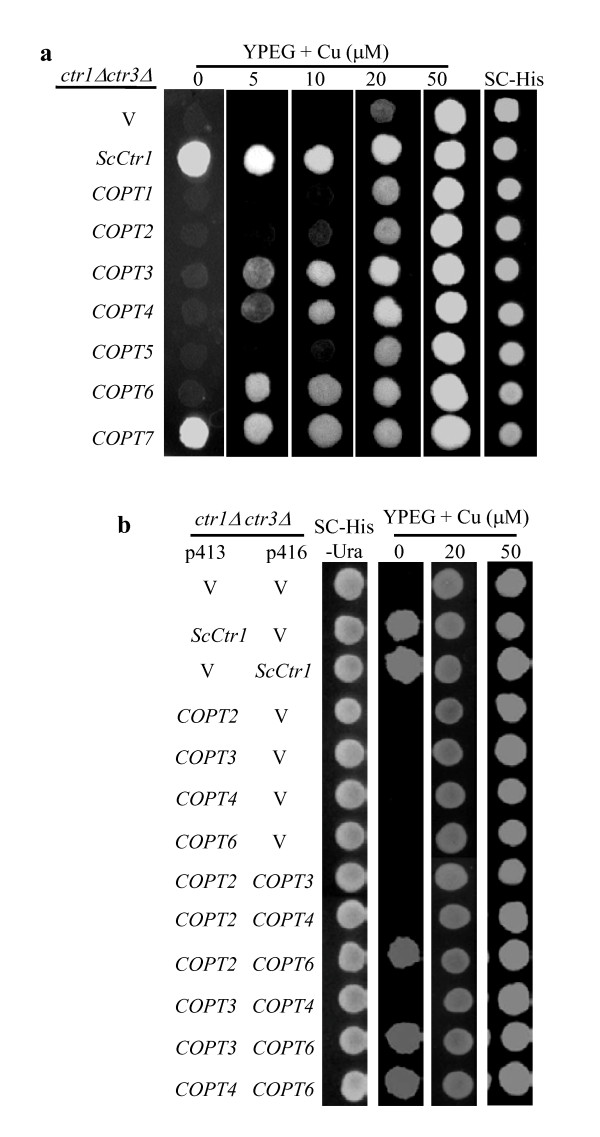
**Functional complementation of *S. cerevisiae ctr1Δctr3Δ*mutant (MPY17) by expression of rice *COPTs***. Yeast *ScCtr1 *was used as positive control. Empty vector (V) was used as negative control. Transformants were grown in SC-His or SC-His-Ura medium to exponential phase and then spotted onto ethanol/glycerol (YPEG)-selective media supplemented with 5 to 50 μM CuSO_4 _or without supplementation of CuSO_4 _(0). (a) Expression of *COPTs *alone. (b) Coexpression of *COPTs*. The p413 and p416 are yeast expression vectors.

A recent study has revealed that rice COPT1 and COPT5 alone or cooperatively cannot complement the phenotype of yeast *S. cerevisiae *mutant MPY17, which lacked the functions of Ctr1 and Ctr3 for Cu uptake; however, rice COPT1 and COPT5 in cooperation with another rice protein XA13 can mediate a low-affinity Cu transport in yeast MPY17 mutant and the three proteins also cooperatively mediate Cu transport in rice plants [29]. To investigate the potential roles of the other five rice *COPT *genes in Cu transport, these genes were expressed separately in MPY17 mutant under the control of the constitutive yeast glyceraldehide-3-phosphate dehydrogenase (GPD) gene promoter [39]. All the transformants were able to grow on SC-His, demonstrating the presence of the expression vector or target gene (Figure 5a). The lack of growth of MPY17 cells containing empty vector (negative control) was restored when transformed with yeast *ScCtr1 *(positive control) in the selective ethanol/glycerol (YPEG) medium without supplementation of Cu. Rice COPT7 but not rice COPT2, COPT3, COPT4, or COPT6 could replace the function of ScCtr1 in yeast cells. Expression of rice COPT7 alone could efficiently complement the phenotype of MPY17 mutant in the medium without supplementation of Cu as compared to the positive control (Figure 5a).

Rice COPT6 could efficiently complement MPY17 phenotype in the medium supplemented with 5 μM Cu compared to the growth of MPY17 cells transformed with COPT7 and the positive control, in the same medium (Figure 5a). Rice COPT3 and COPT4 could also complement MPY17 growth in the medium supplemented with 5 μM Cu, but with a relatively low efficiency. Like rice COPT1 and COPT5, rice COPT2 could not complement MPY17 growth in the medium supplemented with 5 μM Cu compared to the negative control. To determine whether expression of XA13 could enhance the ability of COPT2, COPT3, COPT4, or COPT6 to rescue the yeast mutant, we coexpressed these proteins with XA13. Coexpression of COPT2, COPT3, COPT4, or COPT6 with XA13 could not complement the phenotype of MPY17 (Additional file, Figure S1), which is consistent with our previous results that these COPT proteins can not interact with XA13 [29].

However, coexpression of COPT2, COPT3, or COPT4 with COPT6 efficiently complemented the phenotype of MPY17 in the media without supplementation of Cu as compared to the positive control, while coexpression of COPT2-COPT3, COPT2-COPT4, or COPT3-COPT4 could not complement the phenotype of MPY17 (Figure 5b). To ascertain whether the cooperation of these two proteins in Cu uptake in yeast cells was associated with the physical contact of proteins, the interactions of these COPTs were analyzed using the split-ubiquitin system. All seven COPTs could form homodimers in yeast cells (Figure 3). Consistent with the complementation analyses, COPT2, COPT3, or COPT4 could interact with COPT6, in addition to the interaction of COPT1 with COPT5 as reported previously (Figure 3) [29]. No other COPT pairs could form heterodimers in the yeast cells. These results suggest that COPT7 alone and COPT2, COPT3, or COPT4 cooperating with COPT6, respectively, can mediate a highly efficient Cu transport into yeast cells. In addition, COPT3, COPT4, or COPT6 alone appear to mediate low-affinity Cu transport in yeast cells.

**Figure 6:**
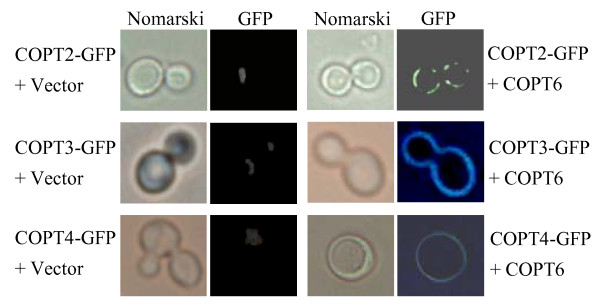
**Localization of COPT2-GFP, COPT3-GFP and COPT4-GFP in yeast MPY17 cells in the presence and absence of COPT6**. MPY17 cells were co-transformed with p413GPD-COPT2-GFP, p413GPD-COPT3-GFP, or p413GPD-COPT4-GFP and p416GPD (empty vector) or p416GPD-COPT6 and grown to log phase on SC-His-Ura. The GFP signal and Nomarski optical images were observed using a fluorescence microscopy.

To ascertain whether the requirement of COPT2, COPT3, or COPT4 with COPT6 to complement MPY17 phenotype was due to COPT6 affecting the localization of these proteins, we examined the localization of these proteins in the MPY17 cells by marking them with GFP. The COPT2-GFP, COPT3-GFP, or COPT4-GFP fusion protein was not or was not largely localized in the plasma membrane of the yeast cells when expressed alone (Figure 6). However, when coexpressed with COPT6, COPT2-GFP, COPT3-GFP, or COPT4-GFP was largely localized in the plasma membrane. These results suggest that COPT6 may function as a cofactor to help the efficient localization of COPT2, COPT3, or COPT4 in the plasma membrane for mediating Cu transport.

### Rice COPTs cannot transport Fe and Zn in yeast

Except for Cu uptake, COPT/Ctr proteins have been reported to be involved in transport other substances [49-51]. To ascertain whether rice COPTs had the capability of transporting other bivalent metal cations in organisms, these *COPT *genes were expressed in yeast *S. cerevisiae *mutants under the control of the *GPD *gene promoter. The *fet3fet4*DEY1453 mutant lacked the Fet3 and Fet4 proteins and was defective in both low- and high-affinity Fe uptake [40]. In the same selective BPDS media with or without supplement of Fe, yeast mutant cells transformed with one of the seven rice *COPTs *showed the same growth pattern as the yeast mutant cells transformed with empty vector (negative control); these cells did not grow in the medium without supplement of Fe (Additional file 1, Figure S2). However, the wild-type yeast strain DEY1457, which was transformed with the empty vector, grew well in the media in all the treatments. These results suggest that none of the rice *COPTs *alone can mediate Fe uptake in yeast.

The *zrt1zrt2*ZHY3 mutant lacked the Zrt1 and Zrt2 proteins for Zn uptake [41,42]. In the selective EDTA media without supplement of Zn, the mutant cells transformed with any one of the rice *COPTs *could not grow as the cells transformed with empty vector (negative control), whereas the wild-type DEY1457 transformed with the empty vector grew well (Additional file 1, Figure S2). These results suggest that rice *COPTs *alone cannot transport Zn in yeast either.

Since coexpression of COPT6 facilitated expression of COPT2, COPT3, or COPT4 in the plasma membrane of yeast cells (Figure 6), we coexpressed each two of the four proteins in *fet3fet4*DEY1453 and *zrt1zrt2*ZHY3 yeast mutants. Coexpression of any two of the four proteins could not complement the phenotypes of the mutants (Additional file 1, Figure S3). These results further support the conclusion that rice COPTs cannot transport Fe and Zn in yeast.

### The expression of COPTs is influenced by multiple factors

**Figure 7:**
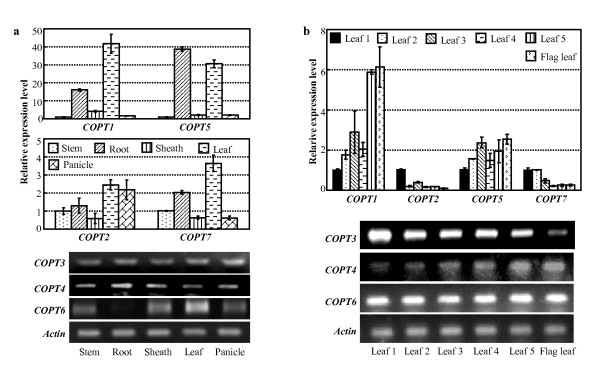
**The tissue-specific and spatiotemporal expression patterns of rice *COPT *genes by qRT-PCR and RT-PCR**. Bar represents mean (3 replicates) ± standard deviation. (a) Expression of *COPTs *in different rice tissues. (b) Expression of *COPTs *in different aged rice leaves in booting-stage plants, which produced six leaves in the main shoot. Leaf 1 was the oldest leaf and flag leaf was the youngest leaf in the plants.

To gain insight into the functions of rice *COPTs*, spatiotemporal and tissue-specific expression patterns of these genes were analyzed by qRT-PCR or RT-PCR (it is difficult to design PCR primers to analyze some *COPT *members by qRT-PCR). *COPT1 *and *COPT5 *showed similar tissue- and development-specific expression patterns. The two genes had a higher level of expression in root and leaf tissues compared to their expression levels in sheath, stem, and panicle (Figure 7a). *COPT4 *showed a higher expression level in root than in other tissues. *COPT1*, *COPT4*, and *COPT5 *had higher expression levels in young leaves than in old leaves, especially for *COPT1 *and *COPT4 *(Figure 7b). *COPT2 *and *COPT3 *had relatively higher expression levels in leaf and panicle than in other tissues and *COPT7 *had relatively higher expression levels in root and leaf than in other tissues; the three genes showed higher expression levels in old leaves compared with young leaves. *COPT6 *was not expressed in root and had a higher expression level in leaf than in other tissues. *COPT6 *showed a constitutive expression pattern in different-aged leaves.

**Figure 8:**
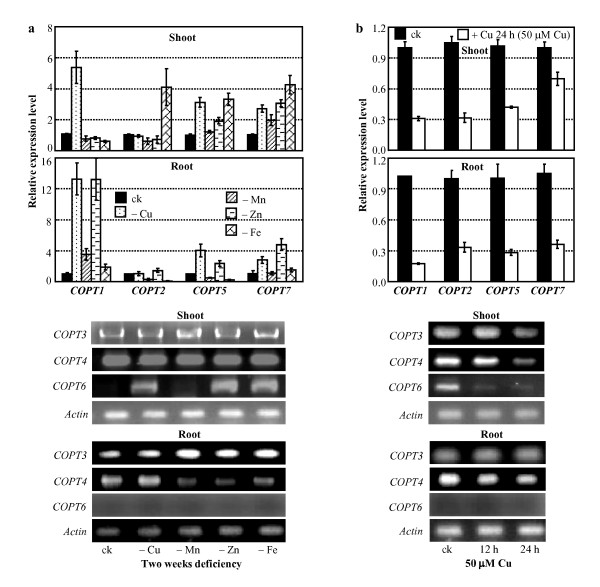
**Expression of *COPTs *in rice was influenced by bivalent cations**. Rice variety Zhonghua 11 at the four-leaf stage grown in hydroponic culture was used for qRT-PCR and RT-PCR analyses. Bar represents mean (3 replicates) ± standard deviation. ck, standard physiological Cu (0.2 mM), Mn (0.5 mM), Zn (0.5 mM), and Fe (0.1 mM). **(**a**) **Expression of *COPTs *was induced at 3 weeks after deficiency of Cu, Mn, Zn or Fe. -Cu, -Mn, -Zn, or -Fe, deficiency of Cu, Mn, Zn, or Fe. **(**b**) **Expression of *COPTs *was suppressed by overdose of Cu.

The expression of rice *COPT1 *and *COPT5 *was influenced by Cu; *COPT1 *and *COPT5 *were induced by Cu deficiency and suppressed by overdose of Cu in both shoot and root tissues [29]. The expression of other five rice *COPTs*, *COPT2*, *COPT3*, *COPT4*, *COPT6*, and *COPT7*, was also influenced by the change of Cu levels (Figure 8). *COPT7 *showed a similar response as *COPT1 *and *COPT5 *to Cu deficiency and overdose (50 μM) in both shoot and root compared to the control plants cultured in the medium containing 0.2 μM Cu. Although *COPT6 *showed constitutive expression in different aged leaves at adult stage (Figure 7b), it had a low level of expression in shoot tissue at seedling stage (Figure 8a). However, *COPT6 *was induced in Cu deficiency and suppressed in Cu overdose in shoot and no *COPT6 *expression was detected either with or without Cu deficiency in root (Figure 8). The expression of *COPT2*, *COPT3*, and *COPT4 *was suppressed in Cu overdose and was not obviously influenced in Cu deficiency in both shoot and root tissues.

Interestingly, other bivalent cations also influenced expression of *COPTs *(Figure 8a). Mn deficiency induced *COPT1 *in root and *COPT3 *and *COPT7 *in shoot, and slightly suppressed *COPT2 *and *COPT4 *in root. Zn deficiency induced *COPT1*, *COPT5*, and *COPT7 *and slightly suppressed *COPT4 *in root and induced *COPT5*, *COPT6*, and *COPT7 *in shoot. Fe deficiency slightly induced *COPT1 *and suppressed *COPT2 *and *COPT5 *in root and induced *COPT2*, *COPT5*, *COPT6*, and *COPT7 *in shoot. No *COPT6 *expression was detected either with or without Mn, Zn, or Fe deficiency in root (Figure 8a).

*Xoo *strain PXO99 induced expression of *COPT1 *and *COPT5*, which encoding proteins interacted with XA13 protein to facilitate *Xoo *infection [29]. To ascertain whether the expression of other *COPTs *was also response to pathogen infection, rice plants were inoculated with different *Xoo *strains. Infection of *Xoo *strain PXO99 could not induce *COPT2*, *COPT3*, *COPT4*, *COPT6*, or *COPT7 *(Additional file 1, Figure S4). Infection of *Xoo *strain PXO66 appeared slightly induced in *COPT1 *and *COPT5*, although the induction was statistically not significant (*P *> 0.05), but not in other *COPTs *(Additional file 1, Figure S5). These results suggest that *COPT *members may function differently in different tissues, different developmental stages, and different environments.

## Discussion

The present results suggest that rice COPT2, COPT3, COPT4, COPT6, and COPT7, which function alone or cooperatively, can replace the roles of ScCtr1 and ScCtr3 for Cu uptake in *S. cerevisiae*. Previous studies have reported that a single plasma membrane-localized COPT/Ctr-type Cu transporter from human (hCtr1), mouse (mCtr1), Arabidopsis (AtCOPT1, 2, 3, and 5), lizard (PsCtr1), Drosophila (DmCtr1A, B, and C), or *Chlamydomonas reinhardtii *(CrCTR1 and CrCTR2) could complement the phenotype of *S. cerevisiae ctr1Δctr3Δ *mutant [13,16,17,23,24,28,52]. The fission yeast (*S. pombe*) SpCtr4 and SpCtr5 form a heteromeric plasma membrane complex to complement the *S. cerevisiae ctr1Δctr3Δ *mutant [19]; coexpression of this complex could also restore the phenotype of *S. pombe ctr4Δctr5Δ *mutant JSY22 [21,53]. Rice COPT1, COPT5, and the MtN3/saliva-type protein XA13, which cooperatively mediate Cu transport in rice, can also complement the phenotype of the *S. cerevisiae ctr1Δctr3Δ *mutant [29]. These results suggest that the *S. cerevisiae ctr1Δctr3Δ *mutant is a valuable model to study the roles of COPT/Ctr-type proteins from different species including rice in Cu transport. Thus, based on the effects of different rice COPTs on the growth of *ctr1Δctr3Δ *mutant cells on the selective media and the expression patterns of these *COPT *genes in response to the variation of Cu concentration in rice, we argue that COPT2, COPT3, COPT4, COPT6, and COPT7 may mediate Cu transport in rice alone or in cooperation with other COPTs.

COPT2, COPT3, or COPT4 may cooperate with COPT6 for Cu transport in different rice tissues except for root. This hypothesis is supported by the following evidence. First, the complementation of the phenotype of *S. cerevisiae ctr1Δctr3Δ *mutant by coexpression of COPT2, COPT3, or COPT4 with COPT6 was consistent with the capability of the physical interaction of COPT2, COPT3, or COPT4 with COPT6 (Figures 3 and 5b). Second, COPT2, COPT3, COPT4, and COPT6 were all expressed in stem, sheath, leaf, and panicle tissues (Figure 7). The similar and tissue-specific expression patterns of *COPT1 *and *COPT5 *(Figure 7) are consistent with their cooperative role in Cu transport in shoot and root reported previously [29]. However, COPT3, COPT4, or COPT6 alone appeared to mediate a low-affinity Cu transport in yeast cells, suggesting that they may be also involved in a low-affinity Cu transport in different rice tissues or rice Cu deficiency alone, although further in planta study is required to examine this hypothesis. This hypothesis is supported by the evidence that *COPT3 *and *COPT4 *had a relatively high level of expression in root, but no expression of *COPT6 *was detected in root; furthermore, the expression of *COPT2*, *COPT3*, and *COPT4 *but not *COPT6 *in leaves was developmentally regulated (Figure 7). In addition, the expression of *COPT6 *but not *COPT2*, *COPT3*, and *COPT4 *was strongly induced by Cu deficiency in rice shoot (Figure 8). The present results also suggest that COPT7 may be capable of mediating Cu transport alone in rice. According to its expression pattern, COPT7 may function in different tissues and also in Cu deficiency.

Except for Cu uptake, human hCtr1 could transport silver (Ag) [51]. This capability of the hCtr1 occurs because Ag(I) is isoelectronic to Cu(I) and thus Ag competes for Cu as the substrate of the hCtr1 [22]. Furthermore, ScCtr1, mCtr1, and hCtr1 could also transport the anticancer drug cisplatin [49,50]. None of the rice COPTs could restore the Fe- or Zn-uptake functions of the yeast mutants (Additional file 1, Figures S2, S3). Overexpressing *COPT1 *or *COPT5 *or suppressing *COPT1 *or *COPT5 *in rice had no influence on Fe, Mn, and Zn contents in rice shoot [29]. These results suggest that rice COPTs may function specifically for Cu transport among bivalent ions like the other COPT/Ctr proteins [32]. That the COPT/Ctr proteins could transport Cu but not Fe, Mn, and Zn may be related to their protein features. The TM domains of COPT/Ctr may form a symmetrical homotrimer or heterotrimer channel architecture with a 9-Å diameter that is suitable only for Cu(I) transport but not other bivalent ions, such as Mn, Zn, or Fe [33].

However, all the rice *COPTs *were transcriptionally activated or suppressed by Fe, Mn, or Zn deficiency (Figure 8a). A balance of the concentrations of Cu with other bivalent ions appears to be associated with their uptake. The yeast *S. cerevisiae *Ctr1 mutants and deletion strains have deficiency in Fe uptake [12]. The high-affinity Fe uptake is influenced by Cu concentration in ascomycetes *C. albicans*; deletion of *CaCTR1 *for Cu uptake results in defective Fe uptake [26]. Excess metals (Fe, Mn, Zn, or cadmium) significantly influenced Cu uptake mediated by human hCtr1 [22]. A yeast *S. pombe ctr6 *mutant displays a strong reduction of Zn superoxide dismutase activity [20]. Thus, further study is required to determine whether other bivalent ion levels influence Cu uptake in rice.

*Xoo *causes bacterial blight, which is one of the most devastating diseases restricting rice production worldwide. Rice COPT1, COPT5, and XA13 cooperate to promote removal of Cu from rice xylem vessels, where *Xoo *multiplies and spreads to cause disease [29]. *COPT1*, *COPT5*, and *Xa13 *can facilitate the infection of *Xoo *strain PXO99 because this bacterium can transcriptionally activate them [29]. The present results suggest that *Xoo*-induced expression of *COPT1 *and *COPT5 *is race specific. Although PXO99 can induce *COPT1 *and *COPT5 *[29], the expression of the two genes was not markedly influenced by *Xoo *strain PXO61 in the infection sites (Additional file 1, Figure S5). Neither PXO61 nor PXO99 influenced the expression of rice *COPT2*, *COPT3*, *COPT4*, *COPT6*, and *COPT7 *in the infection sites, suggesting that these *COPT *genes are not directly involved in the interactions between rice and at least the two *Xoo *strains.

## Conclusion

Like rice COPT1 and COPT5 [29], rice COPT2, COPT3, COPT4, COPT6, and COPT7 also appear to be plasma membrane proteins for they can replace the roles of *S. cerevisiae *plasma membrane-localized ScCtr1 and ScCtr3 for Cu uptake. However, different from COPT1 and COPT5, the other five COPTs may not be directly associated with the rice-*Xoo *interaction. The present results provide tissue and interaction targets of different COPTs for further study of their roles in Cu transport and associated physiological activities in rice.

## Authors' contributions

MY performed functional complementation and gene expression analyses and drafted the manuscript. XL and JX provided biochemical and molecular analysis supports. SW contributed to data interpretation and to writing the manuscript. All authors read and approved the final manuscript.

## Supplementary Material

Additional file 1 **Supplemental tables and figures**. **Table S1**: *PCR primers used for quantitative RT-PCR or RT-PCR assays*. **Table S2**: *PCR primers used for yeast complementation experiments*. **Table S3**: *PCR primers used for protein-protein interaction assays*. **Table S4**: *PCR primers used for protein topology analyses*. **Figure S1**: *Coexpression of rice COPT2, COPT3, COPT4, or COPT6 with Xa13 could not complement S. cerevisiae ctr1Dctr3D mutant (MPY17). *Complementation is indicated by growth on the media with 0, 20, or 50 mM copper (Cu). The p413 and p416 are yeast expression vectors. Yeast *ScCtr1 *and empty vector (V) were used as positive and negative controls, respectively. Transformants were grown in SC-His-Ura medium to exponential phase and spotted onto SC-His-Ura and ethanol/glycerol (YPEG) plates. **Figure S2**: *Analyses of the functions of rice COPTs in Fe-uptake and Zn-uptake mutants of Saccharomyces cerevisiae. *The yeast DEY1457 strain was wild type. Yeast cells diluted in gradient were plated on selective media (OD values: 1, 0.1, 0.01, and 0.001 from left to right). (a) Functional analysis rice COPTs in yeast *fet3fet4*DEY1453 mutant strain, which lacked the Fet3 and Fet4 for Fe uptake. The transformants were spotted onto selective bathophenanthroinedisulfonic acid disodium (BPDS) media with or without supplement of Fe (FeSO4). (b) Functional analysis of rice COPTs in yeast *zrt1zrt*2ZHY3 mutant strain, which lacked the Zrt1 and Zrt2 for Zn uptake. The transformants were spotted onto selective EDTA media with or without supplement of Zn (ZnSO4). **Figure S3**: *Further analyses of the functions of rice COPTs in Fe-uptake and Zn-uptake mutants of Saccharomyces cerevisiae. *The yeast DEY1457 strain was wild type. Yeast cells diluted in gradient were plated on selective media. Empty vector (V) was used as negative control. The p413 and p416 are yeast expression vectors. (a) Functional analysis rice COPT2, COPT3, COPT4, and COPT6 in yeast *fet3fet4*DEY1453 mutant strain, which lacked the Fet3 and Fet4 for Fe uptake. The transformants were spotted onto selective bathophenanthroinedisulfonic acid disodium (BPDS) media with or without supplement of Fe (FeSO4). (b) Functional analysis of rice COPT2, COPT3, COPT4, and COPT6 in yeast *zrt1zrt*2ZHY3 mutant strain, which lacked the Zrt1 and Zrt2 for Zn uptake. The transformants were spotted onto selective EDTA media with or without supplement of Zn (ZnSO4). **Figure S4**: *Infection of Xanthomonas oryzae pv. oryzae strain PXO99 did not influence the expression of COPTs in susceptible rice variety IR24 analyzed by qRT-PCR and RT-PCR. *Bar represents mean (3 replicates) ± standard deviation. Plants were inoculated with PXO99 at booting (panicle development) stage. ck, before infection. **Figure S5**: *The expression of COPTs after infection of Xanthomonas oryzae pv. oryzae strain PXO61 in susceptible rice variety IR24 analyzed by qRT-PCR and RT-PCR. *Bar represents mean (3 replicates) ± standard deviation. Plants were inoculated with PXO61 at booting (panicle development) stage. ck, before infection. Click here for file
