# Supplementary material for: Molecular and functional analyses of COPT/Ctr-type copper transporter-like gene family in rice
Source: BMC Plant Biol. 2011 Apr 21;11:69. doi: 10.1186/1471-2229-11-69 (PMC3103425; doi:10.1186/1471-2229-11-69)
Supplement: Additional file 1 — Supplemental tables and figures. Table S1: PCR primers used for quantitative RT-PCR or RT-PCR assays. Table S2: PCR primers used for yeast complementation experiments. Table S3: PCR primers used for protein-protein interaction assays. Table S4: PCR primers used for protein topology analyses. Figure S1: Coexpression of rice COPT2, COPT3, COPT4, or COPT6 with Xa13 could not complement S. cerevisiae ctr1Dctr3D mutant (MPY17). Complementation is indicated by growth on the media with 0, 20, or 50 mM copper (Cu). The p413 and p416 are yeast expression vectors. Yeast ScCtr1 and empty vector (V) were used as positive and negative controls, respectively. Transformants were grown in SC-His-Ura medium to exponential phase and spotted onto SC-His-Ura and ethanol/glycerol (YPEG) plates. Figure S2: Analyses of the functions of rice COPTs in Fe-uptake and Zn-uptake mutants of Saccharomyces cerevisiae. The yeast DEY1457 strain was wild type. Yeast cells diluted in gradient were plated on selective media (OD values: 1, 0.1, 0.01, and 0.001 from left to right). (a) Functional analysis rice COPTs in yeast fet3fet4DEY1453 mutant strain, which lacked the Fet3 and Fet4 for Fe uptake. The transformants were spotted onto selective bathophenanthroinedisulfonic acid disodium (BPDS) media with or without supplement of Fe (FeSO4). (b) Functional analysis of rice COPTs in yeast zrt1zrt2ZHY3 mutant strain, which lacked the Zrt1 and Zrt2 for Zn uptake. The transformants were spotted onto selective EDTA media with or without supplement of Zn (ZnSO4). Figure S3: Further analyses of the functions of rice COPTs in Fe-uptake and Zn-uptake mutants of Saccharomyces cerevisiae. The yeast DEY1457 strain was wild type. Yeast cells diluted in gradient were plated on selective media. Empty vector (V) was used as negative control. The p413 and p416 are yeast expression vectors. (a) Functional analysis rice COPT2, COPT3, COPT4, and COPT6 in yeast fet3fet4DEY1453 mutant strain, which lacked the Fet3 and Fet4 fo [file 1471-2229-11-69-S1.PDF]

Additional file 1, Table S1. PCR primers used for quantitative RT-PCR or RT-PCR assays

Gene (GenBank accession number)	Primer name	Forward primer (5’-3’)	Reverse primer (5’-3’)	Product size (nt)
COPT1 (GQ387494)	OsCtr1 realF/R	CATGGGCGCCATGAAGTC	GTGAAGAGCACCTCCGAGTTCT	75
COPT2 (HQ833653)	OsCtr2 realF/R	TGCGGCGTGCTGCTAGA	CAAGAGCAGATCCGCACTCA	101
COPT3 (HQ833654)	OsCtr3 1F/1R	GCTCGCCTACCTGGTGATGCT	CGGCTCTGACGATGGATGGA	202
COPT4 (HQ833655)	OsCtr4 1F/1R	CCGCAGCACAAGATGGCGATGA	AGCACGAAGAGGAGGCAGAGGG	128
COPT5 (GQ387495)	OsCtr5 realF/R	GCTGTCTCGCTCGTCATGGT	CGCACACACAAAACATCAACAA	66
COPT6 (HQ833656)	OsCtr6 1F/1R	CGTCCGTCACCATCCTCTTCG	CGGGGCTCCTTCATCCACC	289
COPT7 (HQ833657)	OsCtr7 realF/R	GCCTAGGGTTTGGCTTTGC	ACAAGATCGGGAAACCAAACA	64
Actin (X15865)	Actin-F/R	TGTATGCCAGTGGTCGTACCA	CCAGCAAGGTCGAGACGAA	121

Additional file 1, Table S2. PCR primers used for yeast complementation experiments

Gene (GenBank accession number)	Primer name	Forward primer (5'-3')	Reverse primer (5'-3')	Product size (nt)	Use
COPT1 (GQ387494)	OsCtr1 11F/11RS	CGCGGATCCATGGACATGGGAGGGCAC[a]	CCGGAATTCCTACTAGCAGCAGGCCG[b]	486	Amplifying coding region of *OsCOPT1*
COPT2 (HQ833653)	OsCtr2 6F/6RS	CGCGGATCCATGGCGGACATGGGAAG[a]	CCGGAATTCCTAGCAGCACGCCGCAG[b]	453	Amplifying coding region of *OsCOPT2*
COPT3 (HQ833654)	OsCtr3 4F/4RS	CGCGGATCCATGGCCATGCCCATGCC[a]	CCGGAATTCTTAAGGTTTCGGCTCTG[b]	552	Amplifying coding region of *OsCOPT3*
COPT4 (HQ833655)	OsCtr4 4F/4RS	TAGACTAGTATGAGGGGGATGGGCGA[c]	CCGGAATTCCTAAGTCTTGGATCCATC[b]	555	Amplifying coding region of *OsCOPT4*
COPT5 (GQ387495)	OsCtr5 10F/10RS	CGCGGATCCATGGACATGGGCGGCA[a]	CCGGAATTCCTAGCAGCACACGGGGTC[b]	456	Amplifying coding region of *OsCOPT5*
COPT6 (HQ833656)	OsCtr6 3F/3RS	CGCGGATCCATGATGCACATGAGCTT[a]	CCGGAATTCCTACGCGCAGGCGCAGGGGC[b]	531	Amplifying coding region of *OsCOPT6*
COPT7 (HQ833657)	OsCtr7 4F/4RS	TAGACTAGTATGATGCACATGACCTTC[c]	CCGGAATTCCTAGGCGCAGGCGCAGGG[b]	450	Amplifying coding region of *OsCOPT7*
ScCtr1 (YSCP9642)	ScCtr F/R	CTAGACTAGTATGGAAGGTATGAATA[c]	GCCGATATCTTAGTTATGAGTGAATTT[d]	1221	Amplifying coding region of *ScCtr1*

[a]The underlined nucleotides are the digestion site of *Bam*HI.

[b]The underlined nucleotides are the digestion site of *Eco*RI.

[c]The underlined nucleotides are the digestion site of *Spe*I.

[d]The underlined nucleotides are the digestion site of *Eco*RV.

Additional file 1, Table S3. PCR primers used for protein–protein interaction assays

Gene (GenBank accession number)	Primer name	Forward primer (5’-3’)	Reverse primer (5’-3’)	Product size (nt)	Use
COPT1 (GQ387494)	OsCtr1 5F/5R	GCAATGGCCATTACGGCCAGAAAAATGGACATGGGAGGGCAC[a]	GAATTCGGCCGAGGCGGCCTTGCAGCAGGCCGGGTCGTTCTT[a]	489	Amplifying coding region of *COPT1*
COPT2 (HQ833653)	OsCtr2 BT3F/3R	GCAATGGCCATTACGGCCAGAAAAATGGCGGACATGGG[a]	GAATTCGGCCGAGGCGGCCCAGCACGCCGCAGGCGC[a]	450	Amplifying coding region of *COPT2*
COPT3 (HQ833654)	OsCtr3 BT3F/3R	GCAATGGCCATTACGGCCAGAAAAATGGCCATGCCCATG[a]	GAATTCGGCCGAGGCGGCCGGTTTCGGCTCTGACGA[a]	549	Amplifying coding region of *COPT3*
COPT4 (HQ833655)	OsCtr4 BT3F/3R	GCAATGGCCATTACGGCCAGAAAAATGAGGGGGATGGGC[a]	GAATTCGGCCGAGGCGGCCGTCTTGGATCCATCCGC[a]	552	Amplifying coding region of *COPT4*
COPT5 (GQ387495)	OsCtr5 11F/11R	GCAATGGCCATTACGGCCAGAAAAATGGACATGGGCGGCAATG[a]	GAATTCGGCCGAGGCGGCCTTGCAGCACACGGGGTCGTTCTTG[a]	459	Amplifying coding region of *COPT5*
COPT6 (HQ833656)	OsCtr6 PR3F/3R	CCGGAATTCATGATGCACATGAGCTTCTAC[b]	CCGGAATTCCGCGCAGGCGCAGGGGCTC[b]	528	Amplifying coding region of *COPT6*
COPT7 (HQ833657)	OsCtr7 BT3F/3R	GCAATGGCCATTACGGCCAGAAAAATGATGCACATGACCTTC[a]	GCAGATGGCCGAGGCGGCCGCGCAGGCGCAGGGGTTGTCG[a]	447	Amplifying coding region of *COPT7*

[a]The underlined nucleotides are the digestion site of *Sfi*I.

[b]The underlined nucleotides are the digestion site of *Eco*RI.

Additional file 1, Table S4. PCR primers used for protein topology analyses

Gene (GenBank accession number)	Primer name	Forward primer (5'-3')	Reverse primer (5'-3')	Product size (nt)	Use
COPT2 (HQ833653)	OsCtr2 6F/6R	CGCGGATCCATGGCGGACATGGGAAG [a]	CCGGAATTCGCAGCACGCCGCAGGCG [b]	450	Localization in yeast cells
COPT3 (HQ833654)	OsCtr3 4F/4R	CGCGGATCCATGGCCATGCCCATGCC [a]	CCGGAATTCAGGTTTCGGCTCTGACG [b]	549	Localization in yeast cells
COPT4 (HQ833655)	OsCtr4 4F/4R	TAGACTAGTATGAGGGGGATGGGCGA [c]	CCGGAATTCAGTCTTGGATCCATCCG [b]	552	Localization in yeast cells

[a]The underlined nucleotides are the digestion site of *Bam*HI.

[b]The underlined nucleotides are the digestion site of *Eco*RI.

[c]The underlined nucleotides are the digestion site of *Spe*I.

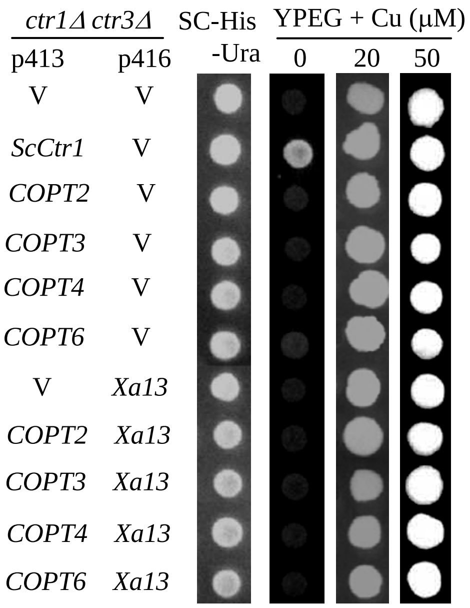

Additional file 1, Figure S1
Coexpression of rice COPT2, COPT3, COPT4, or COPT6 with Xa13 could not complement S. cerevisiae ctr1Δctr3Δ mutant (MPY17). Complementation is indicated by growth on the media with 0, 20, or 50 μM copper (Cu). The p413 and p416 are yeast expression vectors. Yeast *ScCtr1* and empty vector (V) were used as positive and negative controls, respectively. Transformants were grown in SC-His-Ura medium to exponential phase and spotted onto SC-His-Ura and ethanol/glycerol (YPEG) plates.

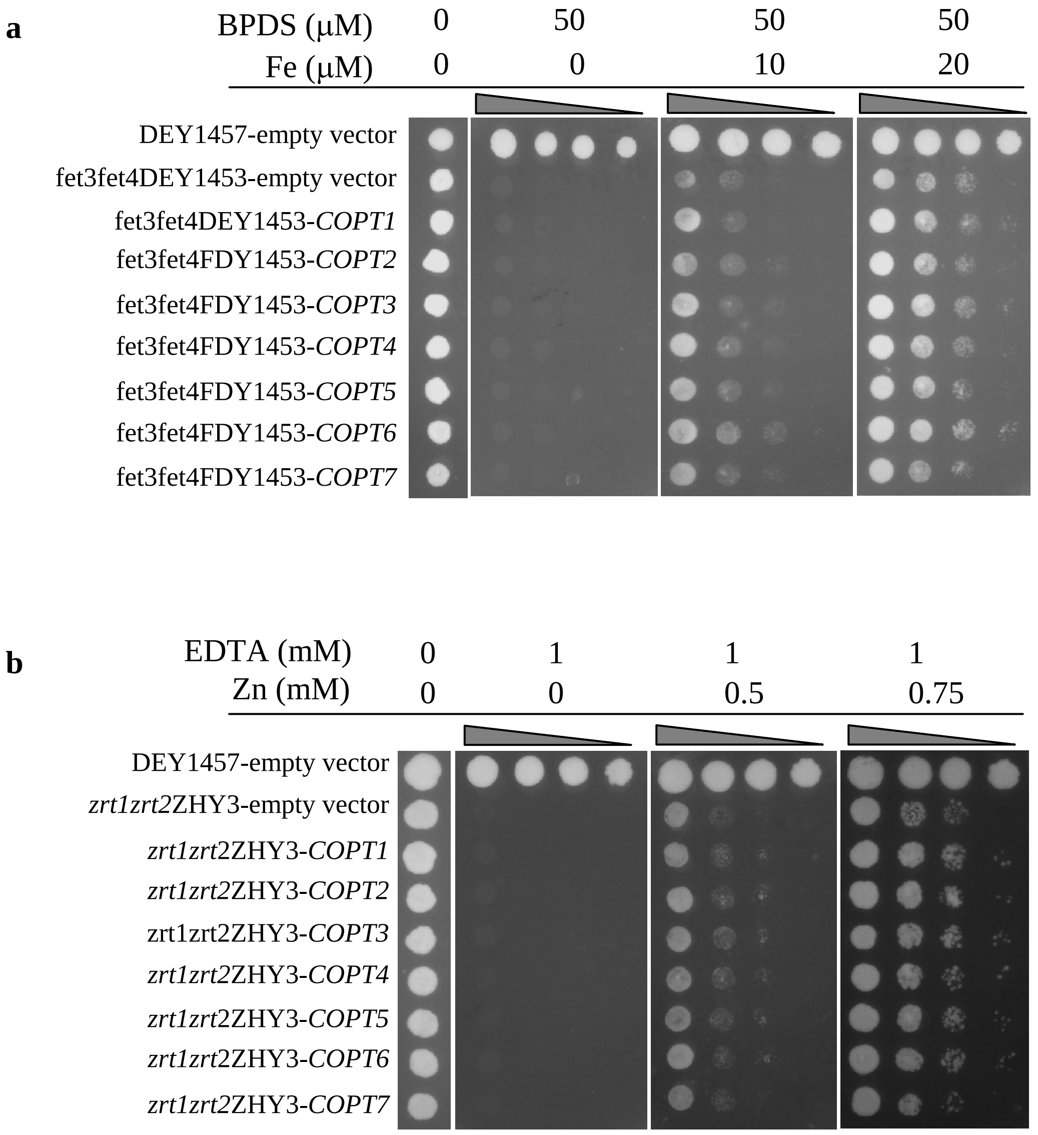

Additional file 1, Figure S2

Analyses of the functions of rice COPTs in Fe-uptake and Zn-uptake mutants of Saccharomyces cerevisiae. The yeast DEY1457 strain was wild type. Yeast cells diluted in gradient were plated on selective media (OD values: 1, 0.1, 0.01, and 0.001 from left to right). (a) Functional analysis rice COPTs in yeast *fet3fet4*DEY1453 mutant strain, which lacked the Fet3 and Fet4 for Fe uptake. The transformants were spotted onto selective bathophenanthroinedisulfonic acid disodium (BPDS) media with or without supplement of Fe ($FeSO_4$). (b) Functional analysis of rice COPTs in yeast *zrt1zrt*2ZHY3 mutant strain, which lacked the Zrt1 and Zrt2 for Zn uptake. The transformants were spotted onto selective EDTA media with or without supplement of Zn ($ZnSO_4$).

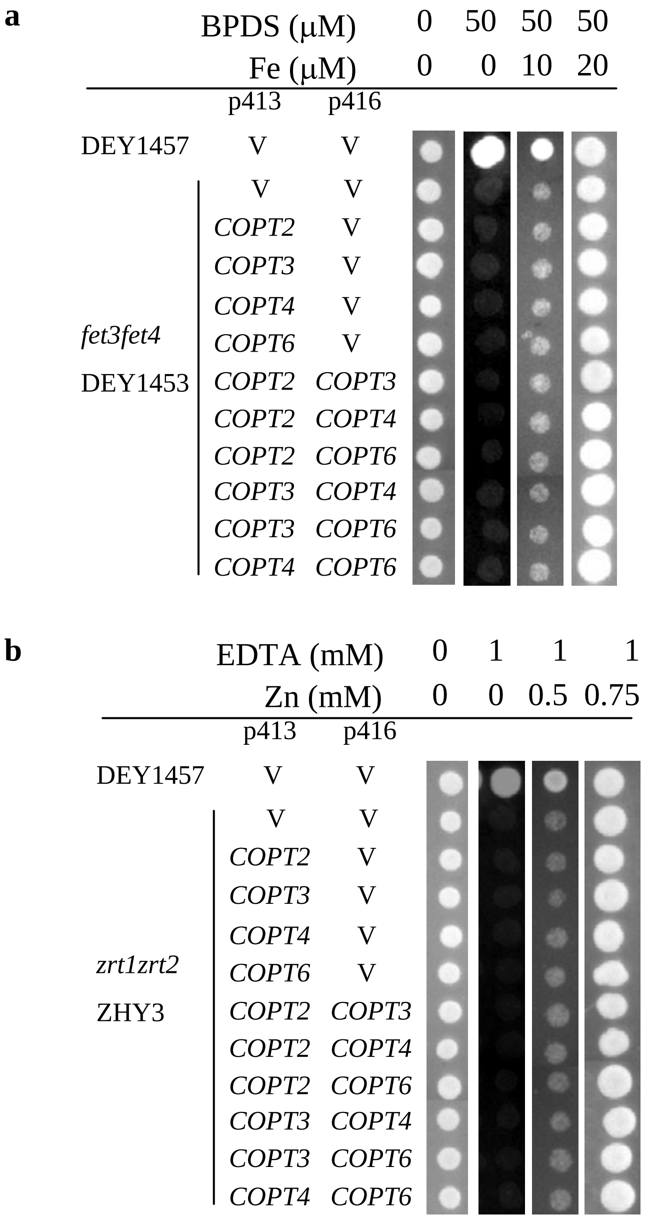

Additional file 1, Figure S3

Further analyses of the functions of rice COPTs in Fe-uptake and Zn-uptake mutants of Saccharomyces cerevisiae. The yeast DEY1457 strain was wild type. Yeast cells diluted in gradient were plated on selective media. Empty vector (V) was used as negative control. The p413 and p416 are yeast expression vectors. (a) Functional analysis rice COPT2, COPT3, COPT4, and COPT6 in yeast *fet3fet4*DEY1453 mutant strain, which lacked the Fet3 and Fet4 for Fe uptake. The transformants were spotted onto selective bathophenanthroinedisulfonic acid disodium (BPDS) media with or without supplement of Fe ($FeSO_4$). (b) Functional analysis of rice COPT2, COPT3, COPT4, and COPT6 in yeast *zrt1zrt*2ZHY3 mutant strain, which lacked the Zrt1 and Zrt2 for Zn uptake. The transformants were spotted onto selective EDTA media with or without supplement of Zn ($ZnSO_4$).

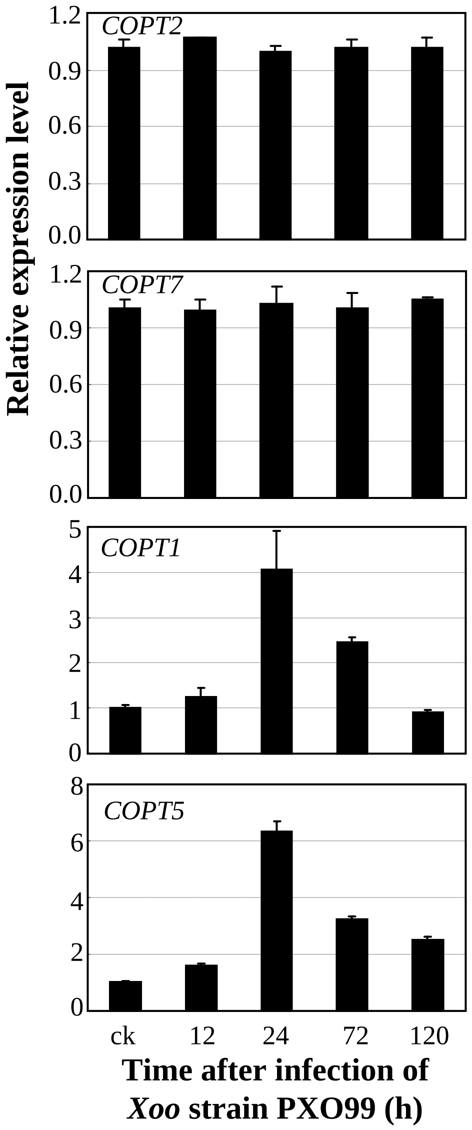

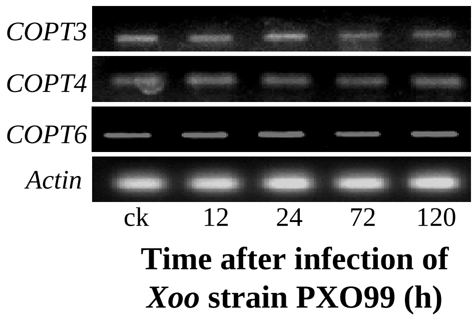

Additional file 1, Figure S4
Infection of Xanthomonas oryzae pv. oryzae strain PXO99 did not influence the expression of COPTs in susceptible rice variety IR24 analyzed by qRT-PCR and RT-PCR. Bar represents mean (3 replicates) ± standard deviation. Plants were inoculated with PXO99 at booting (panicle development) stage. ck, before infection.

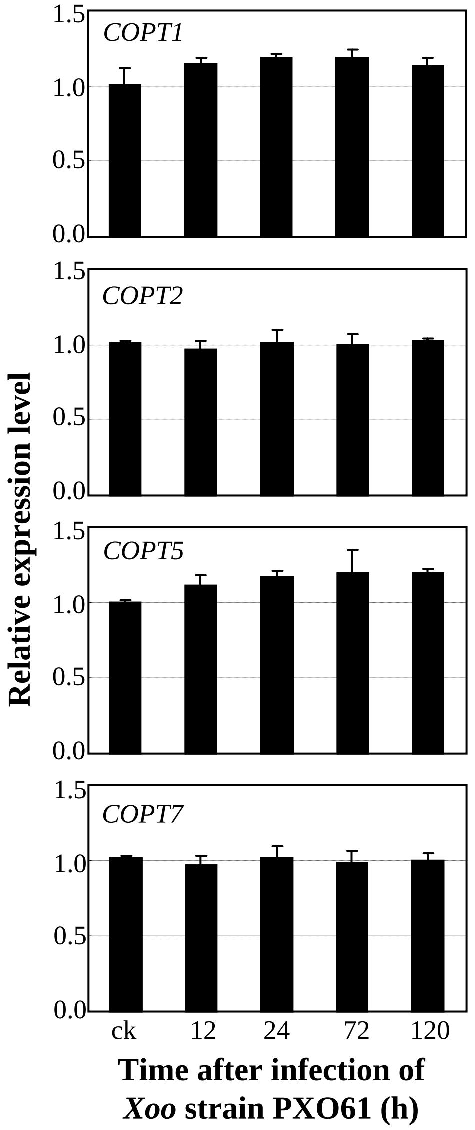

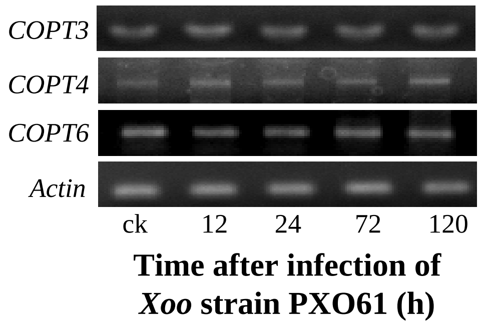

Additional file 1, Figure S5

The expression of COPTs after infection of Xanthomonas oryzae pv. oryzae strain PXO61 in susceptible rice variety IR24 analyzed by qRT-PCR and RT-PCR. Bar represents mean (3 replicates) ± standard deviation. Plants were inoculated with PXO61 at booting (panicle development) stage. ck, before infection.
